# An Investigation of the Healing Efficiency of Epoxy Vitrimer Composites Based on Zn^2+^ Catalyst

**DOI:** 10.3390/polym15173611

**Published:** 2023-08-31

**Authors:** Barbara Palmieri, Fabrizia Cilento, Eugenio Amendola, Teodoro Valente, Stefania Dello Iacono, Michele Giordano, Alfonso Martone

**Affiliations:** 1 Institute of Polymers, Composite and Biomaterials (IPCB), National Research Council of Italy, 80055 Portici, Italy; barbara.palmieri@ipcb.cnr.it (B.P.); teodoro.valente@cnr.it (T.V.); stefania.delloiacono@cnr.it (S.D.I.); michele.giordano@cnr.it (M.G.); alfonso.martone@cnr.it (A.M.); 2 Agenzia Spaziale Italiana (ASI), Via del Politecnico snc, 00133 Roma, Italy

**Keywords:** compression moulding, vitrimer, multifunctional composites, epoxy matrix

## Abstract

The need to recycle carbon-fibre-reinforced composite polymers (CFRP) has grown significantly to reduce the environmental impact generated by their production. To meet this need, thermoreversible epoxy matrices have been developed in recent years. This study investigates the performance of an epoxy vitrimer made by introducing a metal catalyst (Zn^2+^) and its carbon fibre composites, focusing on the healing capability of the system. The dynamic crosslinking networks endow vitrimers with interesting rheological behaviour; the capability of the formulated resin (AV-5) has been assessed by creep tests. The analysis showed increased molecular mobility above a topology freezing temperature (T_v_). However, the reinforcement phase inhibits the flow capability, reducing the flow. The fracture behaviour of CFRP made with the vitrimeric resin has been investigated by Mode I and Mode II tests and compared with the conventional system. The repairability of the vitrimeric CFRP has been investigated by attempting to recover the delaminated samples, which yielded unsatisfactory results. Moreover, the healing efficiency of the modified epoxy composites has been assessed using the vitrimer as an adhesive layer. The joints were able to recover about 84% of the lap shear strength of the pristine system.

## 1. Introduction

The composite industry extensively relies on thermoset materials for their excellent structural performance. However, the challenge lies in their limited recyclability. Thermoset polymers possess a stable cross-linked structure between polymer chains, providing them with high-temperature resistance, thermal and dimensional stability, solvent resistance, and exceptional mechanical properties. Unfortunately, the strong polymer network restricts the long-range molecular mobility required for material flow at high temperatures, impeding their reforming and recycling. Consequently, thermoset polymers and composites are predominantly discarded in landfills after their service life, resulting in a significant environmental impact [1]. Growing environmental consciousness and industrial competitiveness have stimulated the development of repairable and recyclable structural materials to minimise polymer waste and prolong their lifespan [2].

Currently, several methods have been devised to recycle thermoset composites, involving mechanical, chemical, and thermal processes [3]. Mechanical recycling entails shredding procedures to reduce the size of composite components, rendering them recyclable. Thermal recycling involves burning carbon fibre composite materials with or without oxygen to degrade the matrix and extract the fibres [4]. Chemical recycling utilises supercritical fluids and catalytic solutions to separate oligomers obtained from the decomposition of polymeric resin, which can be reused as chemical raw materials along with carbon fibres.

Nevertheless, none of these processes fully align with sustainability requirements [5]. According to the waste management guidelines outlined by the EU [6], waste prevention represents the most effective approach to recycling materials.

To enable the recyclability, weldability, and reparability of thermosetting polymers while retaining their advantageous properties, modifying the polymer network with cleavable or dynamic bonds is a promising approach [7]. The development of Covalent Adaptable Networks (CANs) represents a significant advancement towards producing recyclable epoxy resins suitable for composite components that can be reformed as thermoplastic materials [8,9]. CANs are characterised by covalent crosslinks that possess reversibly dynamic properties under specific external stimuli, such as heat, catalysts, or light [10,11].

CANs can be categorised into dissociative and associative covalent adaptive networks based on the mechanism of reversible chemical bond exchange [12]. Dissociative CANs (dCANs) can be viewed as depolymerisation, involving a reshuffling of the polymeric network that allows for the reworking and reforming of the cross-linked material [10,13]. When subjected to an external stimulus, the entire dCAN network breaks at a faster rate than it reforms, leading to increased polymer mobility due to a reduction in crosslinking density. Once the stimulus is removed, the crosslinking density of the dCAN increases, restoring the mechanical properties to their initial state [14]. One of the extensively studied reaction mechanisms for dCANs is the Diels–Alder reaction [11,15,16,17].

In contrast, associative CANs (aCANs) maintain a constant crosslinking density during the exchange reaction by breaking old bonds and simultaneously generating new bonds. Leibler and colleagues pioneered aCANs in 2011 by employing the well-established transesterification reaction between hydroxyl and ester groups in an anhydride-cured epoxy matrix, resulting in the creation of a reworkable thermosetting network known as vitrimers [18]. During transesterification, the network’s connectivity is altered through exchange reactions, enabling stress relaxation and plastic flow at elevated temperatures without undergoing depolymerisation [19].

Vitrimers exhibit distinct behaviour depending on the temperature conditions. Below the topological freezing transition temperature (T_v_), they behave similarly to traditional thermosets, demonstrating good thermal and mechanical properties. However, above the Tv, vitrimers display the ability to flow like viscoelastic fluids due to the rearrangement of their molecular topology induced by transesterification reactions [8,18].

Among vitrimers, epoxy-based systems utilising transesterification reactions have received significant attention. These systems involve exchange reactions between esters and beta-hydroxyls formed through the reaction of epoxy precursors with suitable acids/anhydrides [8,20]. The incorporation of a catalyst accelerates the transesterification reactions. This leads to topological changes, stress relaxation, and flow in the cross-linked networks without altering the total number of cross-links [21,22].

Various types of catalysts have been explored to facilitate the transesterification reactions and the curing process of epoxy vitrimers [23,24,25]. These catalysts include metal-containing compounds and organocatalysts.

Metal ions, such as zinc cations (Zn^2+^), have been utilised to modify the reactivity of epoxy resin. These Zn^2+^ cations possess a tetracoordinate structure with two neutral and two negatively charged oxygen atoms. In the presence of Zn^2+^, the anhydride group undergoes ligand exchange, leading to the opening of the anhydride ring and the formation of a monoester and a carboxylic acid. Subsequently, this acid reacts with an epoxy ring, resulting in the formation of a diester and the regeneration of a hydroxyl group. This reaction leads to the formation of a β-hydroxyl ester chain [8,20]. Notably, the catalyst Zn(Ac)_2_ plays a pivotal role in accelerating the esterification reaction during the curing process. It has been reported in several studies [8,18,22,26] that Zn(Ac)_2_ is involved in the dynamic transesterification reaction of epoxy-based vitrimer resin.

Yang et al. [27] introduced the transesterification reaction into the anhydride/epoxy system to prepare a repairable hard epoxy, by adding 2%mol of Zinc Acetylacetonate (Zn(acac)_2_). Shi et al. [28] investigated such systems’ capability to be welded and reprocessed. Demongeot et al. [29] investigated the mechanism of action of the catalytic zinc species active in these materials.

Current research is mainly focused on the formulation of epoxy vitrimers and the study of vitrimer static and dynamic properties. The development of carbon material/vitrimer composites is not only for the recycling and reuse of the composite itself but the recycling of carbon materials can also be readily enabled, making such a strategy a win-win and potential way to cope with the thermoset composite waste [30,31]. To address the irreversibility of fatigue, we report here a vitrimeric system, for which the reversal of fatigue damage can be achieved repeatedly by heating the material to above its topology freezing transition temperature. This enables the intermittent healing of fatigue-induced damage, as it accumulates in the vitrimer matrix. Few efforts have addressed fabricating carbon-fibre- or glass-fibre-reinforced composites and exploring their related self-healing and re-use potential. Wu et al. [32] showed that the healing efficiency should be improved by modifying the vitrimer with nanofillers. Delamination is one of the major dangers to the stability and safety of composite structures, hence much attention has been paid to the delamination growth behaviour and interlaminar toughening. Zhao et al. showed that the use of a vitrimer could lead to the recovery of delaminated CFRP [33].

In this study, a commercially available epoxy system, specifically ARALDITE^®^ LY 3508 and ARADUR^®^ 917-1 by Huntsman Corporation, commonly used for carbon-fibre-reinforced polymer (CFRP) manufacturing, was chosen as the base material for vitrimer modification. The resin has been formulated for enhancing the availability of free hydroxyl groups, by keeping the ratio between epoxy and carboxylic acid stoichiometric. The presence of zinc ions embedded within the covalent network promotes transesterification and therefore dynamic crosslinking. The paper investigated the thermomechanical properties of the vitrimer and its carbon fibre composites by comparing them to the unmodified epoxy system. In addition, the chance to repair and reuse has been investigated by attempting to heal CFRP’s delamination and by restoring the joints between CFRP adherends. The joints were able to recover about 84% of the lap shear strength of the pristine system.

## 2. Materials and Methods

### 2.1. Epoxy Vitrimer Formulation and CFRP Manufacturing

The used resin is a mixture of Bisphenol A diglycidyl ether (DGEBA) epoxy resin with an Epoxy Equivalent Weight (EEW) of 196.5 g/eq, tetrahydro-methyl phthalic anhydride (THMPA) curing agent and 2,4,6-tris (dimethyl aminomethyl) phenol as the catalyst, kindly provided by the Huntsman corporation (The Woodlands, TX, USA) with product names ARALDITE^®^ LY 3508, ARADUR^®^ 917-1 and Accelerator 960-1, respectively. Anhydrous Zinc acetate Zn(Ac)_2_ (99.99%) was used as the catalyst, purchased from Merck Sigma-Aldrich (Waltham, MA, USA). All reagents were used without further purification.

An epoxy mixture system, with a stoichiometric ratio of epoxy/acyl equal to 1, named A, has been prepared and the vitrimeric formulation, named AV5, has been obtained by adding 5% of Zn(Ac)_2_ with respect to the total acyl groups.

**Figure 1:**
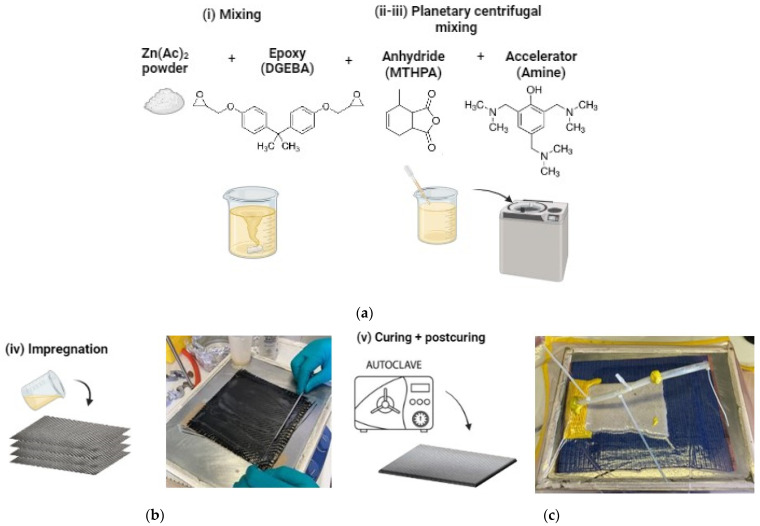
Fabrication procedure of vitrimeric epoxy resin (**a**) and composites (**b**,**c**).

The formulations were prepared following the same procedure, as illustrated in Figure 1a: (i) hand-mixing of epoxy resin ARALDITE^®^ LY 3508 (100 phi) and zinc acetate fine powder (20 μm); (ii) addition of cross-linking anhydride ARADUR^®^ 917-1 (40 phr) and mixing with the planetary centrifugal mixer (THINKY mixer ARV 310, Laguna Hills, CA, USA) under vacuum at room temperature; (iii) addition of the Accelerator 960-1 (3 phr) and further mixing as previous step obtaining a homogeneous mixture. Finally, samples were cast in Teflon moulds, then cured for 1 h 30 min at 120 °C and post-cured for 2 h at 140 °C in the oven.

Carbon fibre (CF)-reinforced composite plates have been manufactured by using both the standard epoxy resin (A) and the vitrimeric epoxy resin (AV5), obtaining CF-A and CF-AV5 panels. Carbon fibre fabric 3k T300 Twill 2 × 2 (Toray) has been impregnated with resin (Figure 1b). Carbon fibre composites were manufactured by hand lay-up, following the lamination sequence [0–90/90–0] and consolidated in the autoclave for 1 h 30 min at 120 °C and then post-cured 2 h at 140 °C in the oven (Figure 1c).

### 2.2. Experimental Characterisation

Thermogravimetric analysis (TGA) (TA Instruments Q500, New Castle, DE, USA) was conducted to evaluate the polymer thermal stability range, according to ASTM E1131. Measurements were performed in an inert atmosphere, using nitrogen gas, with a temperature ramp of 10 °C/min from room temperature to 800 °C. The weight loss is evaluated at 600 °C.

The thermal properties of the polymer were investigated by differential scanning calorimetry (DSC) using Discovery DSC of TA Instruments. Each specimen was heated and cooled twice from 0 to 250 °C at a rate of 10 °C/min under a nitrogen atmosphere. About 10 mg samples were encapsulated in aluminium pans before measurements. The glass transition temperature (T_g_) and enthalpy of the reaction were extracted from the DSC curves, according to ASTM D3418.

Dynamic mechanical analysis (DMA) was performed with a Dynamic Mechanical Analyzer Q800 from TA Instruments in the Single Cantilever mode (SC). Samples of a rectangular shape 25 mm in length, 5.5 mm in width and about 2.5 mm in thickness are tested. Both the behaviour of the polymers and the composites, with temperatures between 30 and 180 °C, were investigated by considering a heating rate of 3 °C/min, a strain amplitude of 15 μm, and a frequency of 1 Hz. Data are elaborated according to the ASTM D790 standard for the flexural behaviour of unreinforced and reinforced plastics [34].

Tensile tests were performed with an Instron 68TM-50 universal testing apparatus. Dogbone samples of 2.5 mm thickness were tested at room temperature with a displacement rate of 2 mm/min. Data are elaborated according to the ASTM D638 standard for plastic tensile strength tests [35].

Creep tests were performed using the DMA Q800 from TA Instruments equipped with the Tension Film (TF) clamp. Rectangular specimens of 10 mm × 5.5 mm × 2 mm were tested. Tests are performed at different temperatures from 70 °C to 245 °C, with 25 °C incremental steps to evaluate the sample strain variation from the glassy to the rubbery state.

The Plane-Strain Fracture Toughness of polymers has been investigated according to ASTM D5045. Single-Edge Notch-Bend (SENB) specimens of dimensions 15 × 3.50 × 1.75 cm^3^ were prepared. The crack length, *a*, of dimension 0.45 W < *a* < 0.55 W were carried out using a blade. Tests were performed using the DMA Q800 from TA Instruments equipped with the 3-point bending clamp and a displacement speed of 0.5 mm/min. The sample nominal dimensions are 15 × 3.50 × 1.75 cm^3^.

For Mode I fracture, composite specimens were prepared according to ASTM D5528 std with dimensions 125 × 25 × 2 mm^3^, and an initial crack of 65 mm. The edges of Double cantilever beams (DCBs) samples were painted with a white correctional fluid to improve crack visibility, and markings were added to track crack growth to the nearest millimetre. Steel loading blocks were glued to the ends of the sample beams using a cyanoacrylate adhesive. The bonding surface of the specimen has been lightly scrabbed with sandpaper and then wiped clean with methylethylketone (MEK) to remove any contamination.

The mode I fracture toughness (*G_Ic_*, kJ/m^2^) of a DCB is calculated according to Equation (1):

(1) $$G_{IC}=\frac{3P\delta }{2ba}$$

where *P* (N) is the load, *δ* (mm) is the load point displacement, *b* (mm) is the sample width, and *a* (mm) is the crack length at fracture. The test beam was loaded in displacement control mode (2 mm/min), from the loading blocks until the crack front propagated about 20 mm, before unloading.

The Mode II interlaminar fracture toughness, *G_IIc_* (kJ/m^2^), is given by Equation (2):

(2) $$G_{IIC}=\frac{9a_{0}^{2}P\delta }{2b({2L}^{3}+3a_{0}^{3})}$$

where *P* (N) is the critical load, *a_0_* (mm) is the initial crack length, *δ* (mm) is the load point displacement, *L* (mm) is the half span, and *b* (mm) is the beam width. Three specimens with dimensions of 160 × 25 × 2 mm^3^ and an initial crack of 45 mm were tested according to ASTM D7905 std [36] on a 3-point bending fixture. End notch flexure (ENF) beams were set such that the crack tip was a fixed distance from one of the support rollers (*a*_0_ = 30 mm) and loaded at 1 mm/min.

## 3. Results

### 3.1. Thermal Characterisation

**Figure 2:**
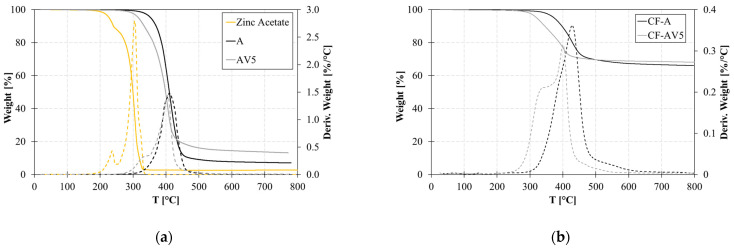
TGA curves on conventional epoxy and vitrimer and zinc acetate (**a**) and composites (**b**). Continuous lines refer to the weight loss and dotted lines to the derivative weight vs temperature.

Figure 2 depicts the thermal degradation up to 800 °C of the standard and modified epoxy resin and on the composites. The presence of zinc acetate in the vitrimeric polymer (AV5) leads to a lower onset temperature of thermal degradation. In fact, it is observed that zinc acetate begins to degrade around 230 °C and had its main degradation at 300 °C. System A experiences a 5% weight loss at 358 °C, which reduces to 315 °C with the addition of zinc acetate. Also, an increase in the residue at 600 °C is found in AV5. This is attributed to the char formation of zinc acetate powder, which exhibits a 2.7% residue at 600 °C.

This behaviour is also shown by composites, where the thermal degradation of the vitrimeric composite CF-AV5 occurs at a lower temperature compared to CF-A. The actual carbon fibre content of CF-A and CF-AV5, reduced by the resin char, is 64% and 63%, respectively.

**Figure 3:**
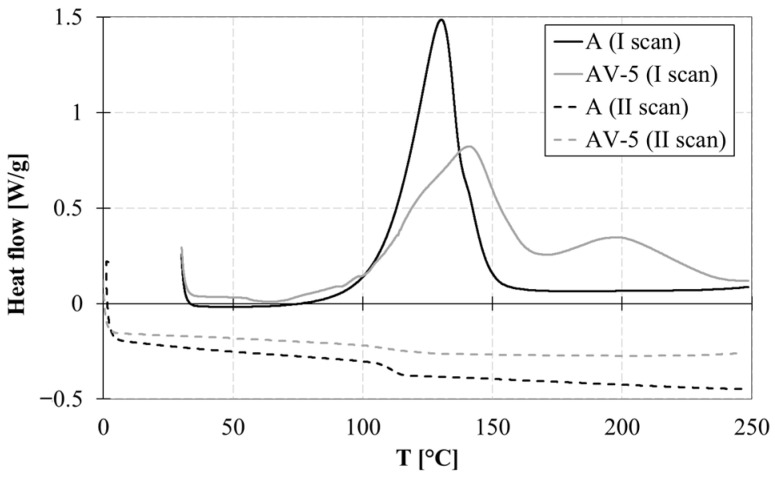
DSC curves of samples A and AV5: I and II scan.

Figure 3 illustrates the curing behaviour and T_g_ of the epoxy and modified epoxy systems. In the case of the modified system (AV5), two exothermic peaks are observed around 140 °C and 180–230 °C. The first peak corresponds to the curing esterification reaction, while the second peak represents the homomeric ring-opening polymerisation of excessive epoxy groups. The presence of zinc acetate affects the primary cross-linking reaction and triggers a secondary reaction between 180 and 230 °C.

During the second heating ramp (Figure 3), the absence of an exothermic peak indicates complete polymer conversion. The T_g_ of the cross-linked sample is influenced by the catalyst content: system A exhibits a T_g_ of 111.2 °C; in the case of the vitrimeric formulations AV5, the T_g_ decreases due to a lower cross-linking density. Composites CF-A and CF-AV5 exhibited a slightly higher value of T_g_ of 116.2 °C and 107.3 °C, respectively.

Table 1 provides a summary of the thermal behaviour of the analysed samples, characterised through TGA and DSC experiments.

### 3.2. Static and Dynamic Mechanical Characterisation

**Figure 4:**
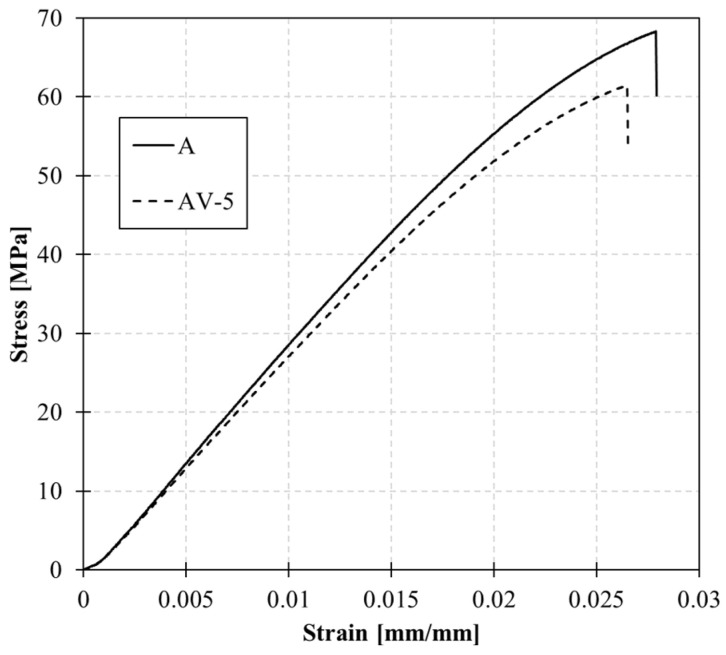
Tensile test on samples A and AV5.

Stress–strain curves of the tensile tests conducted on the samples are depicted in Figure 4 and the values of ultimate stress, ultimate strain, and elastic modulus calculated within the elastic range (0 to 0.005 mm/mm) are reported in Table 2. The incorporation of zinc within the molecular topology affects the stiffness of the system, as evidenced by a decrease in Young’s modulus and strength. The catalyst content influences the mobility of the system, as reflected in the reduction in the T_g_.

**Figure 5:**
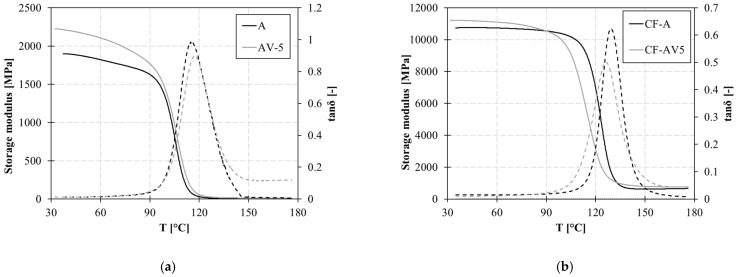
DMA curves for: (**a**) neat polymer (A and AV5) and (**b**) composite (CF-A and CF-AV5). Continuous lines refer to the storage modulus and dotted lines to the tanδ.

The viscoelastic behaviour of polymers with temperatures up to 180 °C is reported in Figure 5. DMA experiments were conducted within the linear viscoelastic deformation range to determine the T_g_ and elastic modulus of the cross-linked samples. In this case, with the addition of zinc acetate, no significant variation is observed in the T_g_.

The introduction of the Zn^2+^ catalyst results in a slight increase in the storage modulus compared to system A. Similarly, the dissipation capacity, measured by the loss modulus (E″), also shows an increase with the addition of zinc acetate.

Improvements of 17% and 29% are found for the storage modulus (E′) and E″, respectively. As a result, the loss factor also increases by 10%.

At a higher temperature of 170 °C, the loss moduli of the vitrimeric system significantly increase compared to the non-vitrimeric system (Table 3). This indicates the higher mobility of the polymer at high temperatures induced by the catalyst.

The improvement in the viscoelastic behaviour of the vitrimeric polymer is reflected in the composite. Sample CF-AV5 shows a higher storage modulus at room temperature compared to CF-A. Most importantly, the effect of zinc acetate on molecular mobility is also shown at high temperatures for the composite. Indeed, at 170 °C, the dissipation capacity of the vitrimeric composite is improved.

### 3.3. Isothermal Creep Test

Creep experiments provide explicit and reliable evidence of the vitrimeric behaviour of the resin, which involves thermoreversible rearrangements of cross-linking when a constant load is applied. For each step, samples are isothermally held for five minutes, and then a constant stress of 0.1 MPa (for A and AV5) and 10 Mpa (for CF-AV5) was applied for 45 min.

As anticipated, the sample with the non-vitrimeric formulation (A) exhibited stable behaviour. It showed a negligible increase in strain at higher temperatures, demonstrating typical thermosetting behaviour. Even at temperatures exceeding the T_g_, no molecular flow was observed.

**Figure 6:**
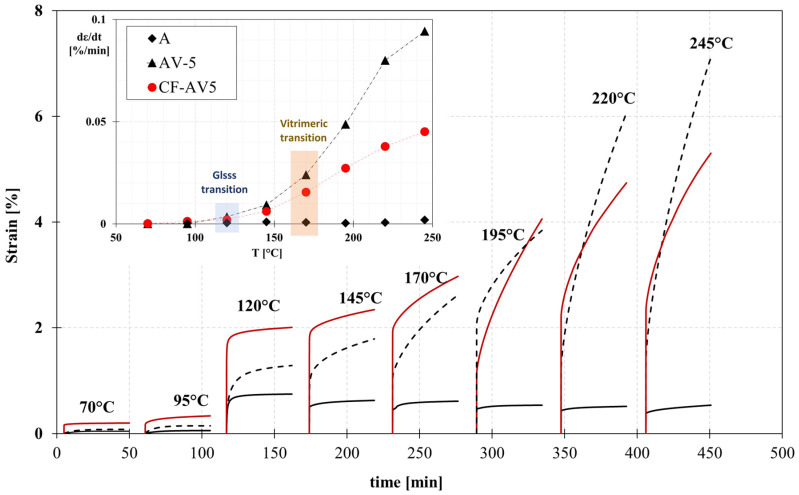
Creep curves at different temperatures of systems A (continuous black line), AV5 (dotted black line), and CF-AV5 (continuous red line).

In contrast, the vitrimeric formulations displayed a significant increase in strain under a constant load at elevated temperatures above T_g_. The presence of metallic ions, such as zinc acetate, acted as strong catalysts for the ester interchange reaction, leading to noticeable creep in samples subjected to tensile loads at various temperatures, as depicted in Figure 6. The influence of the zinc acetate content was also observed, with a significant molecular flow occurring at temperatures above 170 °C, which represents the topological freezing transition temperature (T_v_).

### 3.4. Fracture Behaviour

**Figure 7:**
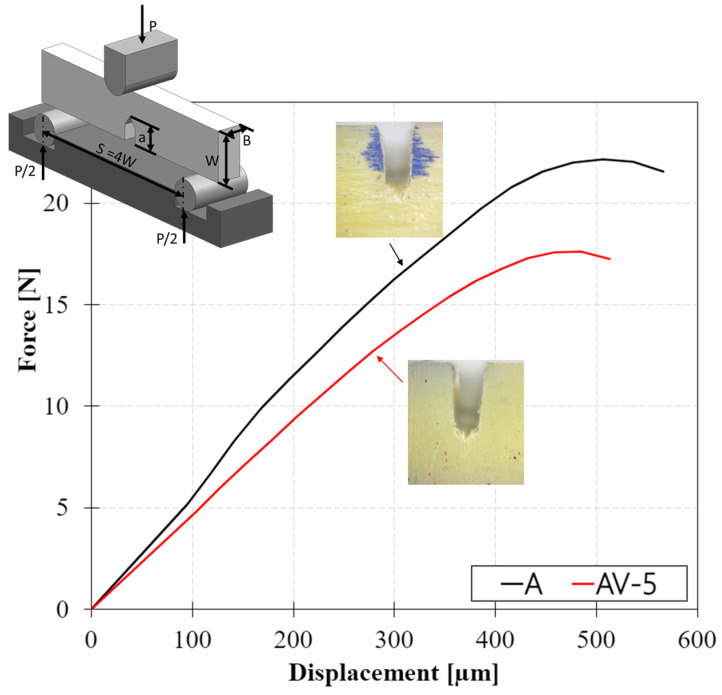
Force displacement curves for A and AV5 for critical-stress-intensity factor.

The force–displacement curves of SENB tests conducted on standard and vitrimeric epoxy resins are reported in Figure 7. The mechanical response of the specimens was linear and elastic up to fracture.

The test setup is shown in Figure 7. The Plane-Strain Fracture Toughness, *G_Q_*, in units of kJ/m^2^, which represents the resistance of a material to fracture in a neutral environment in the presence of a sharp crack under severe tensile constraint, is calculated according to Equation (3):

(3) $$G_{Q}=\frac{U}{B\cdot W\cdot \phi }$$

where *U* (kJ) is the corrected energy, *B* (mm) is the thickness, *W* (mm) is the width, and ϕ is the energy calibration factor.

Therefore, the fracture toughness of the polymer has been computed according to ASTM D5045 (Equation (3)). Two main parameters describe the fracture toughness of a material: K_1C_, the critical stress intensity factor, and the fracture toughness *G_Q_*. The K_1C_^,^ value, which is the energy necessary to separate two surfaces during the fracture propagation, is obtained by the peak load of the sample before breaking, while *G_Q_* is calculated as the area beneath the load–displacement curve. The results of the SENB experiments are reported in Table 4; epoxy resin A showed a fracture toughness *G_Q_* equal to 3.13 J/m^2^, which slightly reduces in the case of vitrimeric resin to 2.85 J/m^2^.

**Figure 8:**
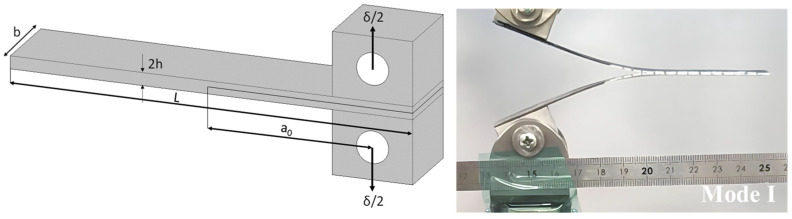
Mode I DCB testing configuration.

**Figure 9:**
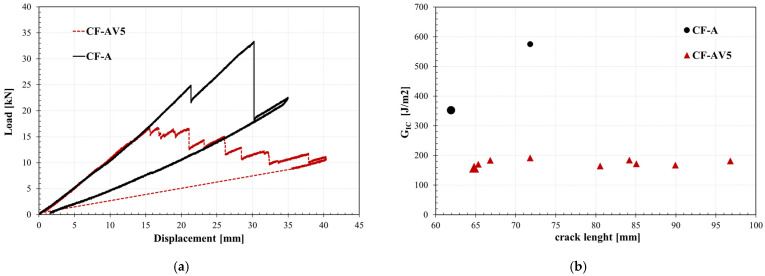
Results of DCB test: (**a**) Load-displacement curve; (**b**) Mode I fracture toughness (*G_Ic_*).

The load–displacement curves of DCB tests, of which the testing configuration is reported in Figure 8, are depicted in Figure 9a. The curves show the relation between the applied load and displacement for the vitrimeric carbon fibre composite and the standard systems. Initially, the two systems behave equally. In the case of the vitrimeric system, the initiation of crack growth occurs prematurely compared to the non-vitrimeric epoxy system. Also, a noticeable difference in the crack propagation pattern is shown: the crack growth is much smoother than the reference sample, meaning that the fracture propagation is slower and more controlled.

The mode I fracture toughness, calculated according to Equation (1), is reported in Figure 9b as a function of crack length. The load curves show a linear increase with a similar gradient until the crack starts to propagate, which indicates a similar elastic coefficient of all laminates. Laminates with the standard matrix gained a higher load than the epoxy vitrimer CFRP, suggesting a better interface adhesion between epoxy and carbon fibres.

The first value of *G_Ic_* (on initiation) indicates the energy required for the initial crack extension and is defined as the point of the sudden decrease in load. The increase in *G_Ic_* with the crack length is mainly due to the fibre bridging that happens on the fracture surface and is indicated as *G_Ic_* upon propagation [37].

Vitrimeric composites showed lower values of both *G_Ic_* on initiation and propagation compared to the non-vitrimeric epoxy. The higher value of *G_Ic_* is consistent with the reported results of fracture toughness for the bulk standard and vitrimeric systems. However, the crack propagation of the vitrimeric system is much higher in the vitrimeric composite.

**Figure 10:**
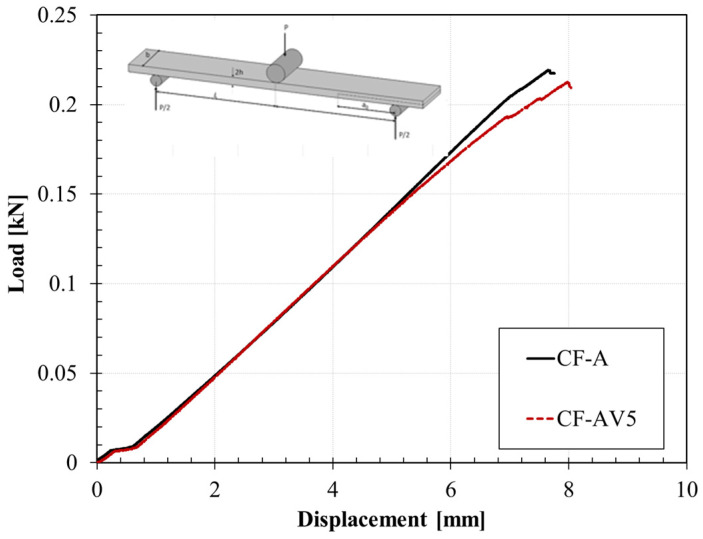
Load–displacement curves of ENF tests.

A slight reduction in the Mode II fracture toughness (*G_IIC_*, Equation (2)) is found in the case of the vitrimeric composite, being 463 J/m^2^ in the case of non-vitrimeric composite and 459 J/m^2^ in the case of vitrimeric composite. Figure 10 shows the load–displacement curves for standard and vitrimeric carbon fibre reinforced systems. Table 5 summarises the fracture toughness results of the Mode I and Mode II tests.

## 4. Discussion

### 4.1. Epoxy Vitrimer Structure and Its Effect on the Dynamic Properties

In the associative bond exchange reaction, the overall crosslink density remains constant; the crosslinks are broken only when new ones are created without resulting in a loss of crosslinks at the previous position [38]. The copolymerisation between epoxy and anhydride in the presence of 960-1 is the main reaction during the curing process of the base system. The anhydride rings (TMPHA) are opened by the hydroxyl present within the accelerator to form carboxylic groups, which react with the epoxy groups forming an ester and a new hydroxyl which contribute to the formation of further esters and lead to a polymer chain including polyester. Here, the copolymerisation of epoxy and anhydride-forming ester bonds is dominant, and the effects of secondary reactions should not be considered. A crosslinked network structure containing a large number of ester bonds was formed by the curing reaction of anhydride and epoxy groups [29,39,40].

**Figure 11:**
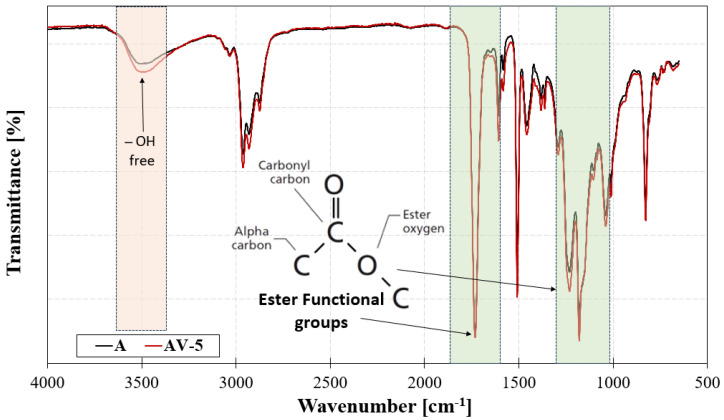
FT-IR spectra of A and AV5 systems. Orange and green boxes highlight the –OH groups and ester groups signals.

Infrared Spectroscopy (FT-IR) (Frontier MIR/NIR Perkin-Elmer spectrometer, Waltham, MA, USA) was adopted to investigate the presence of the typical ester peaks. Spectra were acquired on both sides of the samples. A spectral window range, corresponding to the remarkable peaks of epoxy resin [41], from 650 cm^−1^ to 4000 cm^−1^ was adopted. Figure 11 shows the FT-IR spectra of the standard and vitrimeric epoxy resins.

The spectra exhibit a peak at ≈3500 cm^−1^ due to the presence of the free hydroxyl group, which relies on the reaction between the carboxylic acid and epoxy ring promoting the esterification. The availability of –OH groups facilitates transesterification in the case of the AV5 system where metal catalysts (Zn^2+^) have been provided within the network. Furthermore, the spectra also exhibit strong absorption peaks in the 1650–1800 cm^−1^ and 1000–1250 regions, corresponding to the ester groups containing one C=O bond and two C–O bonds. These results indicate that the DGEBA epoxy group opened to crosslink with the curing agent (acid anhydrides, MHTPA) and formed an ester-bond-based crosslinked network, which is the base for the transesterification exchange reaction. The peak at 1732 cm^−1^ represents the carbonyl stretch, and for saturated esters in general these peaks fall from 1755 to 1735 [42]. The second peak, labelled at 1235 cm^−1^, is from the stretching of the C–O bond to the left of the ester oxygen, which is attached to the carbonyl carbon, and involves the stretching of the alpha carbon-carbonyl carbon C–C bond. The third peak at 1100 depends on the second C–O bond in the ester, which is the one on the right of the bond. For statured esters in general, the O–C–C stretch is present from 1100–1030 cm^−1^.

The presence of an ester group makes the crosslinked network a little more flexible, therefore the fracture toughness of the epoxy system is enhanced with respect to a conventional amine-cured epoxy, while the polyesters induce a plasticising effect. In fact, based on the results of the SENB tests, as reported in Table 4, the K_IC_ was 2.24 MPa √m for the bare epoxy/anhydride resin which is very high compared to amine-cured epoxies [43].

**Figure 12:**
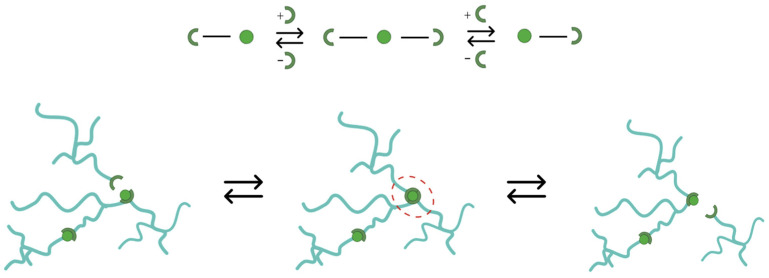
Exchange reaction scheme and related topological changes. The dashed rec circle highlights that the exchange reaction does preserve the average functionality of crosslinks, and depolymerization in the intermediate step is not required.

The preparation of cross-linked systems featuring semi-flexible molecular structures facilitates topological interchange reactions. The reactivity of interchange linkages and thermomechanical properties could be balanced by modifying the amount of catalyst in the formulation [27]. The transesterification reaction is activated by the presence of Zn^2+^ ions at temperatures beyond the vitrimeric temperature (T_v_), leading to a topological rearrangement. The ester bond exchange can then be activated at high temperatures (above a topology freezing temperature, T_v_) when a transesterification catalyst (Zn^2+^, in the current case) is administered into the crosslinked network (Figure 12).

The presence of Zn(Ac)_2_ catalyst affects the thermal behaviour of epoxy vitrimers; the DSC thermograms of the uncured samples have shown a reduction in the T_g_ of the vitrimeric resins compared to the standard epoxy system. Additionally, the reaction enthalpy increases when the zinc acetate content is added to the system.

The T_g_ obtained from DMA analyses is higher than those from DSC, primarily due to the delayed response during temperature scanning in the DMA test caused by the larger sample size [44].

The tanδ of vitrimeric systems at 170 °C is significantly higher than the corresponding conventional formulations. It increases with the increasing temperature, resembling the typical behaviour of thermoplastic polymers [45].

By observing the strain rate (dε/dt), defined as the slope of the strain versus time in the last 5 min of the creep test, the modified systems (AV5 and CF-AV5) clearly show a strong increase in the strain rate with respect to the conventional system which should not flow (Figure 6). Therefore, the addition of the catalyst induces a molecular flow; indeed, the strain rate increases as the temperature increases due to the mobility of polymeric chains promoted by the transesterification reactions.

**Figure 13:**
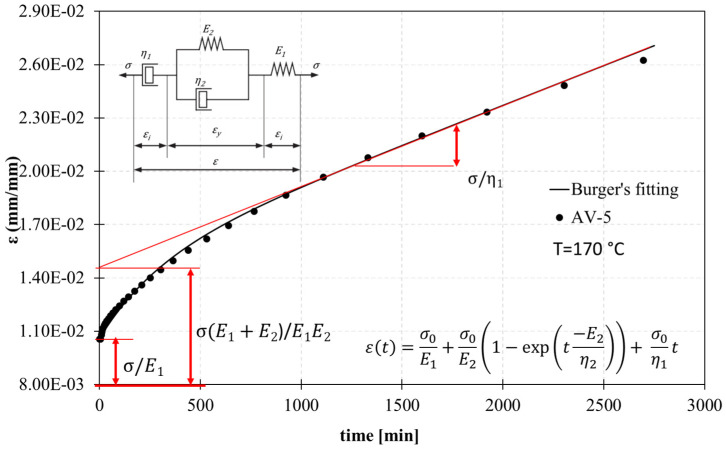
Best fitting of creep data for the AV5 system at 170 °C using Burger’s viscoelastic model. The red lines indicate the different components of the Burger’s viscoelastic model equation.

To assess the vitrimer-like nature of epoxy and investigate the flow at high temperatures, creep tests have been performed and analysed using a theoretical model. Several theoretical models are available to analyse the experimental creep curves. In this study, the Burger model has been implemented, which combines the Maxwell and Kelvin–Voigt elements [46]. According to Burger’s model, the total strain in the creep is the result of instantaneous deformation and the deformation at primary and secondary stages (Figure 13).

The amount of total strain is given by Equation (4):

(4) $$\varepsilon \left(t\right)=\varepsilon_{i}+\varepsilon_{y}$$

where ε(t) is the total strain obtained during the creep test at a particular time t, $\varepsilon_{i}$ is the instantaneous deformation, and $\varepsilon_{y}$ is the delay elastic deformation of the Kelvin–Voigt element. The Equation (4) can be rewritten as:

(5) $$\varepsilon \left(t\right)=\frac{\sigma_{0}}{E_{1}}+\frac{\sigma_{0}}{E_{2}}\left(1-exp(t\frac{{-E}_{2}}{\eta_{2}})\right)+\frac{\sigma_{0}}{\eta_{1}}t\;$$

where *σ*_0_ is the applied stress; *E*_1_ represents the modulus of longitudinal elasticity at the initial deformation which can be recovered once the stress is removed (Maxwell spring); the constant *η*_1_ is the coefficient of dynamic viscosity, and identifies the constant rate of stationary creep; *E*_2_ is the stiffness of the amorphous chain/retardant elasticity and is represented by the spring in the Kelvin–Voigt unit; and *η*_2_ is the viscosity of the Kelvin–Voigt unit and the ratio between *η*_2_/*E*_2_ is the retardation time (*τ*). To determine the values of the parameters (*E*_1_, *E*_2_, *η*_1_, and *η*_2_), curve fitting of the experimental creep curves with Burger’s model was performed.

**Figure 14:**
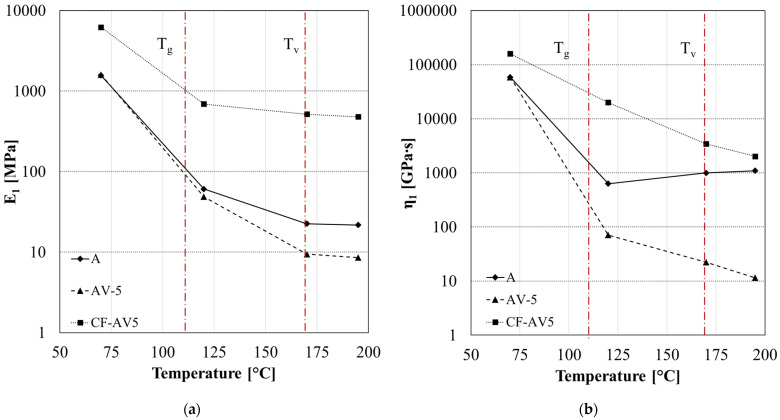
Fitting parameters of creep analysis for system A, AV5, and CF-AV5: (**a**) elastic modulus *E*_1_ and (**b**) permanent viscous flow *η*_1._ Red dotted lines indicate the T_g_ and T_v_.

**Figure 15:**
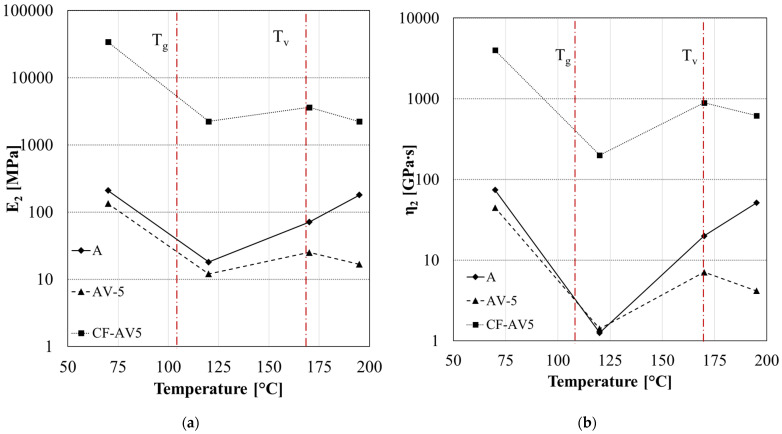
Fitting parameters of creep analysis for system A, AV5, and CF-AV5: (**a**) retardancy elasticity *E*_2_ and (**b**) viscosity *η*_2_. Red dotted lines indicate the T_g_ and T_v_.

Figure 14 and Figure 15 report the fitting parameters for the creep curves in the case of A-system, its vitrimeric modification, and the carbon-fibre-reinforced samples at different temperatures (70 °C, 120 °C,170 °C, 195 °C). The temperature was chosen according to the different regimes: glassy (below T_g_), viscoelastic solid (above T_g_, below T_v_), and liquid viscoelastic (above T_v_).

The elastic modulus E_1_ (Figure 14a) decreases with increasing temperature due to the molecular rearrangement. Unlike the standard epoxy system (A), the vitrimeric systems (AV5 and CF-AV5), above T_v_, exhibit a further reduction in the elastic modulus resembling the thermoplastic-like behaviour. The carbon fibre reinforcement only affects the values of the elastic modulus without modifying the viscoelastic behaviour.

Starting from 170 °C, the vitrimeric epoxy becomes less stiff and the polymer starts to flow, resulting in a progressive decrease in the elastic modulus.

*η*_1_ represents the irrecoverable part of the creep deformation, and it indicates the residual strain left in the material. Like *E*_1,_ this parameter decreases with the temperature until the T_g_, due to the greater mobility of the molecular chains (Figure 14b). In the case of A systems, it remains constant with increasing temperature due to the thermosetting nature of the material. In the case of vitrimeric system AV5 and its composite (CF-AV5), *η*_1_ decreases with the temperature, while the standard epoxy achieves a constant value due to the absence of flow. The presence of the zinc catalyst enables flow (low *η*_1_ parameter) and increases at a higher temperature; above T_v_, the raising of the exchange reaction (transesterification) induces further molecular flow. The presence of carbon fibre reinforcement inhibits the flow, as shown in Figure 14b. As observed, the CF-AV5 system shows a higher *η*_1_ value, which reproduces a system able to recover the applied strain. Even increasing the temperature leads to a system being unable to gain irreversible deformation. Only above the T_v_ has a slight decrease in flow parameter been observed.

The parameters *E*_2_ (Figure 15a) and the *η*_2_ (Figure 15b) represent the retardancy elasticity and viscosity, respectively, and are associated with the stiffness and viscous flow of amorphous polymer chains. A similar dependency of the retardancy elasticity and viscosity on the temperature has been observed for both the neat vitrimer and the reinforced one. Below the glass transition (T < T_g_), both parameters decrease due to the greater energy absorbed by the active polymer chains, and the viscous slippage of the molecules becomes easier to achieve. In the temperature range between the glass transition and vitrimeric temperatures (T_g_ < T < T_v_), the *E*_2_ and *η*_2_ increase with the temperature due to the more significant orientation of polymer chains along the creep loading direction, which results in an orientational hardening. Above the vitrimeric temperature (T > T_v_), a further reduction in retardancy parameters is observed only in the case of vitrimeric systems (AV5 and CF-AV5) due to the molecular flow induced by the transesterification reaction [47].

### 4.2. Influence of Reinforcement on the Repairability of CFRP Vitrimers

**Figure 16:**
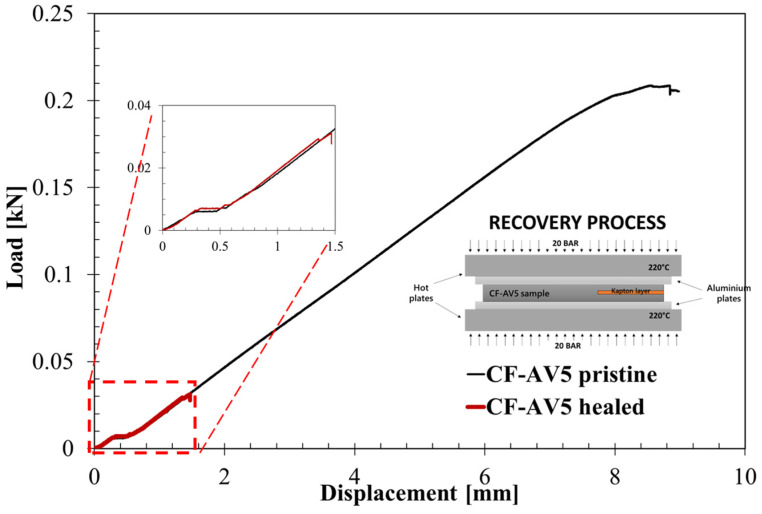
Comparison of healed ENF sample vs. pristine one.

Previous analysis showed that the CF reinforcement limited the ability of the system to flow. An investigation of the reparability of delamination has been carried out on previously tested ENF samples. Fracture surfaces have been stacked and the healing has been set via a hot press and compression moulded at 20 bar and 220 °C for 1 h (see inset in Figure 16). The healing conditions have been chosen according to previous experience on the neat vitrimer.

Even if the samples, macroscopically, looked perfectly joined, the mechanical strength of the specimen is not satisfactory. The load–displacement curves (Figure 16) showed that the healed sample recovered its initial stiffness (see magnification inset within Figure 16), since the curve perfectly reproduces the first run. Nevertheless, the sample reproduced an extremely brittle behaviour with a sudden failure at a load 50 times lower than the pristine strength. A rationale for the latter behaviour could be the detained flux, which is congruent with the creep analysis where CF-AV5 reproduced a high viscosity coefficient (*η*_1_, see Figure 14), indicating a system unable to achieve irreversible deformation. Therefore, the resin flow is limited by the fibres requiring a different strategy to promote the healing of the system.

### 4.3. Use of Vitrimer as Adhesive Layer: Reassembly of Lap Shear Joints

**Figure 17:**
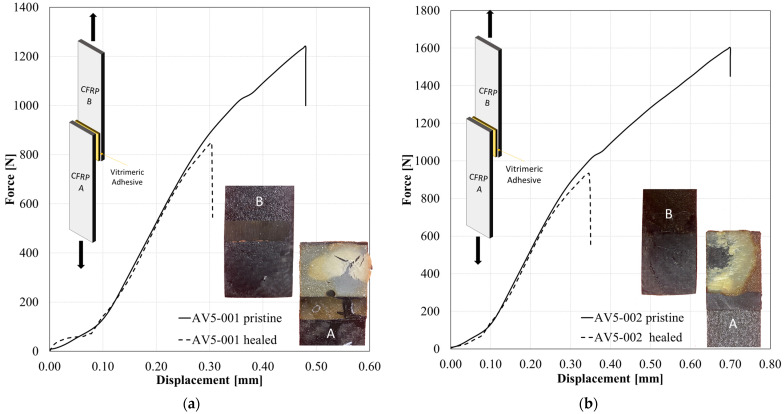
Lap shear test of AV5 as an adhesive on CFRP substrates: (**a**) samples AV5-001 and (**b**) AV5-002. The letter A and B indicates the two adherends of the joint, respectively.

The potential of using epoxy vitrimer materials as the recoverable adhesive was also demonstrated in this work. The AV5 vitrimer was employed as an adhesive layer between two CFRP adherends in a single lap shear configuration (Figure 17) and cured. The lap shear strength (*LSS*, MPa) of the joints was calculated as the ratio between the maximum load (*F_max_*, N) and the total overlap area (*A_LSS_*, mm^2^), as given by Equation (6):

(6) $$LSS=\frac{F_{max}}{A_{LSS}}$$

The results obtained are reported in Table 6. Unlike the traditional epoxy adhesive, the AV5 system is designed on a bond-exchangeable crosslinked network structure, and the joint should be restored by a proper thermomechanical stimulus. According to the previously described procedure for the bare vitrimer, the two substrates (cohesive failure) were successfully re-bonded together by heating at 200 °C (above the T_v_) for 1 h by keeping the contact using a constant load. The restored joints were tested again by following the same test procedure in order to assess the healing capability.

The efficiency of the LSS healed joints is defined as the percent of recovered strength according to the Equation (7):

(7) $$\eta \;[\%]=\frac{P_{healed}}{P_{pristine}}\cdot 100$$

The average recovery of the joints is 84% of the pristine.

It is worth noting that, in some cases (i.e., sample AV5-002), the healing efficiency was significantly lower (61%). In that case, by analysing the fracture surface (inset in Figure 17b), the flow generated during the healing process led the resin to squeeze out from the joint, contributing to the formation of a misalignment between substrates and resulting in a reduced *LSS* value.

## 5. Conclusions

The role of reinforcement on fibre-reinforced composites made using epoxy vitrimers is crucial for assessing the capability of the system to be repaired or to be re-used. Here, we investigated the healing efficiency of epoxy vitrimer composites based on the transesterification reaction made by adding a metallic catalyst (zinc ions) within the covalent network.

The formulated resin is characterised by a stoichiometric epoxy-to-acyl group ratio in order to have enough free hydroxyl groups available for esterification. This result was also confirmed through the FT-IR analysis; the addition of the zinc ions made the system prone to transesterification and activated a flow with a proper thermal stimulus. The thermomechanical results and creep test demonstrated that the catalyst induces the vitrimeric behaviour.

The presence of the reinforcement inhibited the ability of the system to flow; the analysis of the creep data showed that the topological freezing regime is extended to higher temperatures with respect to the bare polymer, resulting in a system unable to efficiently recover the failure. The fracture mechanics performances of carbon fibre composites made using the vitrimer have been assessed by investigating the Double Cantilever and End Notched tests, showing a drop of the Mode I fracture toughness and a slight reduction for Mode II with respect to the conventional epoxy system (no catalyst).

The repairability of the vitrimeric CFRP has been investigated by attempting to recover the delaminated samples and by further testing. The results were not satisfactory.

The vitrimer has been employed as a structural adhesive layer between CFRP adherends. The broken samples were then resorted using a previously defined procedure and tested again. The potential use of the vitrimeric resin as a re-bondable adhesive has been investigated by LSS. The efficiency of the healed sample reached 84%.

## Figures and Tables

**Table 1:** Results of TGA and DSC analyses.

Description	Residue at 600 °C [wt%]	CF Actual Content [wt%]	T_g, DSC_ [°C]
A	7.8	-	111.2
AV5	14.5	-	105.1
CF-A	67.2	64.4	116.2
CF-AV5	68.9	62.6	107.3

**Table 2:** Ultimate tensile stress and strain and elastic modulus for A and B systems.

	Elastic Modulus [MPa]	Ultimate Strain [mm/mm]	Ultimate Stress [Mpa]
A	2704 ± 5	0.028 ± 0.002	68.3 ± 0.7
AV5	2596 ± 8	0.027 ± 0.003	61.5 ± 0.6

**Table 3:** Results of DMA analysis at 35 °C and 170 °C.

		@35 °C			@170 °C		
	T_g, DMA_	E′	E″	tan δ	E′	E″	tan δ
	[°C]	[Mpa]	[Mpa]	[-]	[Mpa]	[Mpa]	[-]
A	105.6 ± 1.1	1899	22.1	0.0116	5.14	0.02	0.0459
AV5	108.8 ± 0.9	2221	28.5	0.0128	12.08	1.43	0.1180
CF-A	123.6 ± 0.8	10,717	166.5	0.0155	649.4	6.33	0.0097
CF-AV5	115.3 ± 1.2	11,210	111.2	0.0099	762.6	33.46	0.0439

**Table 4:** Fracture toughness results for SENB tests.

Description	*G_Q_*_,_ [kJ/m^2^]	K_Ic,_ [Mpa √m]
A	3.13 ± 0.7	2.24 ± 0.3
AV5	2.85 ± 0.8	2.01 ± 0.4

**Table 5:** Fracture toughness Mode I and Mode II.

Description	*G_Ic_*_, initiation_ [J/m^2^]	*G_Ic_*_, propagation_ [J/m^2^]	*G_IIc_* [J/m^2^]
CF-A	352 ± 22	574 ± 30	463 ± 35
CF-AV5	158 ± 10	184 ± 15	459 ± 16

**Table 6:** Results of LSS tests conducted using AV5 as hot melt adhesive.

Description	*LSS* [MPa]	Healing Efficiency [%]
AV5_001	2.31	-
AV5_001_Healed	1.94	84%
AV5_002	2.41	-
AV5_002_Healed	1.48	61%

## Data Availability

Not applicable.

## References

[1] Witik R.A., Teuscher R., Michaud V., Ludwig C., Månson J.-A.E. (2013). Carbon Fibre Reinforced Composite Waste: An Environmental Assessment of Recycling, Energy Recovery and Landfilling. Compos. Part A Appl. Sci. Manuf..

[2] de Luzuriaga A.R., Martin R., Markaide N., Rekondo A., Cabañero G., Rodríguez J., Odriozola I. (2016). Epoxy Resin with Exchangeable Disulfide Crosslinks to Obtain Reprocessable, Repairable and Recyclable Fiber-Reinforced Thermoset Composites. Mater. Horizons.

[3] Kumar S., Krishnan S. (2020). Recycling of Carbon Fiber with Epoxy Composites by Chemical Recycling for Future Perspective: A Review. Chem. Pap..

[4] Palmieri B., Borriello C., Rametta G., Iovane P., Portofino S., Tammaro L., Galvagno S., Giordano M., Ambrosio L., Martone A. (2023). Investigation on Stress Relaxation of Discontinuous Recycled Carbon Fiber Composites. J. Mater. Eng. Perform..

[5] Prinçaud M., Aymonier C., Loppinet-Serani A., Perry N., Sonnemann G. (2014). Environmental Feasibility of the Recycling of Carbon Fibers from CFRPs by Solvolysis Using Supercritical Water. ACS Sustain. Chem. Eng..

[6] Malinauskaite J., Spencer N. (2017). Waste Prevention and Technologies in the Context of the EU Waste Framework Directive: Lost in Translation?. Eur. Energy Environ. Law Rev..

[7] Memon H., Wei Y., Zhu C. (2022). Recyclable and Reformable Epoxy Resins Based on Dynamic Covalent Bonds—Present, Past, and Future. Polym. Test..

[8] Capelot M., Unterlass M.M., Tournilhac F., Leibler L. (2012). Catalytic Control of the Vitrimer Glass Transition. ACS Macro Lett..

[9] Imbernon L., Norvez S. (2016). From Landfilling to Vitrimer Chemistry in Rubber Life Cycle. Eur. Polym. J..

[10] Dello Iacono S., Martone A., Pastore A., Filippone G., Acierno D., Zarrelli M., Giordano M., Amendola E. (2017). Thermally Activated Multiple Self-Healing Diels-Alder Epoxy System. Polym. Eng. Sci..

[11] van den Tempel P., van der Boon E.O., Winkelman J.G.M., Krasnikova A.V., Parisi D., Deuss P.J., Picchioni F., Bose R.K. (2023). Beyond Diels-Alder: Domino Reactions in Furan-Maleimide Click Networks. Polymer (Guildf)..

[12] Zhang H., Cui J., Hu G., Zhang B. (2022). Recycling Strategies for Vitrimers. Int. J. Smart Nano Mater..

[13] Wang S., Fu D., Wang X., Pu W., Martone A., Lu X., Lavorgna M., Wang Z., Amendola E., Xia H. (2021). High Performance Dynamic Covalent Crosslinked Polyacylsemicarbazide Composites with Self-Healing and Recycling Capabilities. J. Mater. Chem. A.

[14] Kuang X., Liu G., Dong X., Wang D. (2016). Triple-Shape Memory Epoxy Based on Diels–Alder Adduct Molecular Switch. Polymer.

[15] Amendola E., Palmieri B., Iacono S.D., Martone A. (2023). Thermally Mendable Self-Healing Epoxy Coating for Corrosion Protection in Marine Environments. Materials.

[16] Turkenburg D.H., Fischer H.R. (2015). Diels-Alder Based, Thermo-Reversible Cross-Linked Epoxies for Use in Self-Healing Composites. Polymer.

[17] Fierro G.-P.M., Pinto F., Iacono S.D., Martone A., Amendola E., Meo M. (2017). Monitoring of Self-Healing Composites: A Nonlinear Ultrasound Approach. Smart Mater. Struct..

[18] Montarnal D., Capelot M., Tournilhac F., Leibler L. (2011). Silica-like Malleable Materials from Permanent Organic Networks. Science.

[19] Yue L., Guo H., Kennedy A., Patel A., Gong X., Ju T., Gray T., Manas-Zloczower I. (2020). Vitrimerization: Converting Thermoset Polymers into Vitrimers. ACS Macro Lett..

[20] Wang S., Ma S., Li Q., Xu X., Wang B., Yuan W., Zhou S., You S., Zhu J. (2019). Facile: In Situ Preparation of High-Performance Epoxy Vitrimer from Renewable Resources and Its Application in Nondestructive Recyclable Carbon Fiber Composite. Green Chem..

[21] Denissen W., Winne J.M., Du Prez F.E. (2016). Vitrimers: Permanent Organic Networks with Glass-like Fluidity. Chem. Sci..

[22] Capelot M., Montarnal D., Tournilhac F., Leibler L. (2012). Metal-Catalyzed Transesterification for Healing and Assembling of Thermosets. J. Am. Chem. Soc..

[23] Yang Y., Xu Y., Ji Y., Wei Y. (2021). Functional Epoxy Vitrimers and Composites. Prog. Mater. Sci..

[24] Liu W., Schmidt D.F., Reynaud E. (2017). Catalyst Selection, Creep, and Stress Relaxation in High-Performance Epoxy Vitrimers. Ind. Eng. Chem. Res..

[25] Altuna F.I., Hoppe C.E., Williams R.J.J. (2019). Epoxy Vitrimers with a Covalently Bonded Tertiary Amine as Catalyst of the Transesterification Reaction. Eur. Polym. J..

[26] Yue L., Amirkhosravi M., Gong X., Gray T.G., Manas-Zloczower I. (2020). Recycling Epoxy by Vitrimerization: Influence of an Initial Thermoset Chemical Structure. ACS Sustain. Chem. Eng..

[27] Yang Y., Peng G., Wu S., Hao W. (2018). A Repairable Anhydride-Epoxy System with High Mechanical Properties Inspired by Vitrimers. Polymer.

[28] Shi Q., Yu K., Dunn M.L., Wang T., Qi H.J. (2016). Solvent Assisted Pressure-Free Surface Welding and Reprocessing of Malleable Epoxy Polymers. Macromolecules.

[29] Demongeot A., Mougnier S.J., Okada S., Soulié-Ziakovic C., Tournilhac F. (2016). Coordination and Catalysis of Zn^2+^ in Epoxy-Based Vitrimers. Polym. Chem..

[30] Kamble M., Vashisth A., Yang H., Pranompont S., Picu C.R., Wang D., Koratkar N. (2022). Reversing Fatigue in Carbon-Fiber Reinforced Vitrimer Composites. Carbon.

[31] Wu Y., Wei Y., Ji Y. (2023). Carbon Material/Vitrimer Composites: Towards Sustainable, Functional, and High-Performance Crosslinked Polymeric Materials. Giant.

[32] Wu P., Liu L., Wu Z. (2022). A Transesterification-Based Epoxy Vitrimer Synthesis Enabled High Crack Self-Healing Efficiency to Fibrous Composites. Compos. Part A Appl. Sci. Manuf..

[33] Zhao Y., Zhao M., Wang A., Chang Z., Wang Z., Zhang K. (2023). Experimental Study on the Mode Ι Interlaminar Properties of Self-Healable Vitrimeric CFRP with Various Interfaces. Compos. Part B Eng..

[34] (2002). Test Methods for Flexural Properties of Unreinforced and Reinforced Plastics and Electrical Insulating Materials.

[35] (2015). Standard Test Method for Tensile Properties of Plastics.

[36] (2019). Standard Test Method for Determination of the Mode II Interlaminar Fracture Toughness of Unidirectional Fiber-Reinforced Polymer Matrix Composites.

[37] Pini T., Briatico-Vangosa F., Frassine R., Rink M. (2018). Matrix Toughness Transfer and Fibre Bridging Laws in Acrylic Resin Based CF Composites. Eng. Fract. Mech..

[38] Krishnakumar B., Sanka R.V.S.P., Binder W.H., Parthasarthy V., Rana S., Karak N. (2020). Vitrimers: Associative Dynamic Covalent Adaptive Networks in Thermoset Polymers. Chem. Eng. J..

[39] Zhao W., An L., Wang S. (2021). Recyclable High-Performance Epoxy-Anhydride Resins with DMP-30 as the Catalyst of Transesterification Reactions. Polymers.

[40] Fang M., Liu X., Feng Y., Lu B., Huang M., Liu C., Shen C. (2023). Influence of Zn^2+^ Catalyst Stoichiometry on Curing Dynamics and Stress Relaxation of Polyester-Based Epoxy Vitrimer. Polymer.

[41] Nikolic G., Zlatkovic S., Cakic M., Cakic S., Lacnjevac C., Rajic Z. (2010). Fast Fourier Transform IR Characterization of Epoxy GY Systems Crosslinked with Aliphatic and Cycloaliphatic EH Polyamine Adducts. Sensors.

[42] Xiang Q., Xiao F. (2020). Applications of Epoxy Materials in Pavement Engineering. Constr. Build. Mater..

[43] Daelemans L., van der Heijden S., De Baere I., Muhammad I., Van Paepegem W., Rahier H., De Clerck K. (2015). Bisphenol A Based Polyester Binder as an Effective Interlaminar Toughener. Compos. Part B Eng..

[44] Chekanov Y., Arrington D., Brust G., Pojman J.A. (1997). Frontal Curing of Epoxy Resins: Comparison of Mechanical and Thermal Properties to Batch-Cured Materials. J. Appl. Polym. Sci..

[45] Suslick B.A., Hemmer J., Groce B.R., Stawiasz K.J., Geubelle P.H., Malucelli G., Mariani A., Moore J.S., Pojman J.A., Sottos N.R. (2023). Frontal Polymerizations: From Chemical Perspectives to Macroscopic Properties and Applications. Chem. Rev..

[46] Ward I.M., Sweeney J. (2012). Mechanical Properties of Solid Polymers.

[47] Lorandi N.P., Cioffi M.O.H., Shigue C., Ornaghi H.L. (2018). On the Creep Behavior of Carbon/Epoxy Non-Crimp Fabric Composites. Mater. Res..

